# Lactobacilli Attenuate the Effect of *Aggregatibacter actinomycetemcomitans* Infection in Gingival Epithelial Cells

**DOI:** 10.3389/fmicb.2022.846192

**Published:** 2022-05-04

**Authors:** Manuela R. Bueno, Karin H. Ishikawa, Gislane Almeida-Santos, Ellen S. Ando-Suguimoto, Natali Shimabukuro, Dione Kawamoto, Marcia P. A. Mayer

**Affiliations:** ^1^Department of Microbiology, Institute of Biomedical Sciences, University of São Paulo, São Paulo, Brazil; ^2^Department of Stomatology, School of Dentistry, University of São Paulo, São Paulo, Brazil; ^3^Department of Immunology, Institute of Biomedical Sciences, University of São Paulo, São Paulo, Brazil

**Keywords:** gingival epithelial cells, *Aggregatibacter actinomycetemcomitans*, probiotics, periodontitis, immune response

## Abstract

Probiotics may be considered as an additional strategy to achieve a balanced microbiome in periodontitis. However, the mechanisms underlying the use of probiotics in the prevention or control of periodontitis are still not fully elucidated. This *in vitro* study aimed to evaluate the effect of two commercially available strains of lactobacilli on gingival epithelial cells (GECs) challenged by *Aggregatibacter actinomycetemcomitans*. OBA-9 GECs were infected with *A. actinomycetemcomitans* strain JP2 at an MOI of 1:100 and/or co-infected with *Lactobacillus acidophilus* La5 (La5) or *Lacticaseibacillus rhamnosus* Lr32 (Lr32) at an MOI of 1:10 for 2 and 24 h. The number of adherent/internalized bacteria to GECs was determined by qPCR. Production of inflammatory mediators (CXCL-8, IL-1β, GM-CSF, and IL-10) by GECs was determined by ELISA, and the expression of genes encoding cell receptors and involved in apoptosis was determined by RT-qPCR. Apoptosis was also analyzed by Annexin V staining. There was a slight loss in OBA-9 cell viability after infection with *A. actinomycetemcomitans* or the tested probiotics after 2 h, which was magnified after 24-h co-infection. Adherence of *A. actinomycetemcomitans* to GECs was 1.8 × 10^7^ (± 1.2 × 10^6^) cells/well in the mono-infection but reduced to 1.2 × 10^7^ (± 1.5 × 10^6^) in the co-infection with Lr32 and to 6 × 10^6^ (± 1 × 10^6^) in the co-infection with La5 (p < 0.05). GECs mono-infected with *A. actinomycetemcomitans* produced CXCL-8, GM-CSF, and IL-1β, and the co-infection with both probiotic strains altered this profile. While the co-infection of *A. actinomycetemcomitans* with La5 resulted in reduced levels of all mediators, the co-infection with Lr32 promoted reduced levels of CXCL-8 and GM-CSF but increased the production of IL-1β. The probiotics upregulated the expression of *TLR2* and downregulated *TLR4* in cells co-infected with *A. actinomycetemcomitans*. *A. actinomycetemcomitans-*induced the upregulation of *NRLP3* was attenuated by La5 but increased by Lr32. Furthermore, the transcription of the anti-apoptotic gene *BCL-2* was upregulated, whereas the pro-apoptotic *BAX* was downregulated in cells co-infected with *A. actinomycetemcomitans* and the probiotics. Infection with *A. actinomycetemcomitans* induced apoptosis in GECs, whereas the co-infection with lactobacilli attenuated the apoptotic phenotype. Both tested lactobacilli may interfere in *A. actinomycetemcomitans* colonization of the oral cavity by reducing its ability to interact with gingival epithelial cells and modulating cells response. However, *L. acidophilus* La5 properties suggest that this strain has a higher potential to control *A. actinomycetemcomitans-*associated periodontitis than *L. rhamnosus* Lr32.

## Introduction

Periodontitis is triggered by a polymicrobial community unbalanced with the host immune response, leading to chronic inflammation (Hajishengallis and Lamont, 2012; Wang, 2015). In periodontal health, the dynamic host–bacteria environment is able to maintain a beneficial commensal microbiota while preventing the emergence of opportunistic pathogens from within these established biofilms (Roberts and Darveau, 2002). However, certain species such as *Porphyromonas gingivalis* and *Aggregatibacter actinomycetemcomitans* play a key role in disrupting the biofilm associated with health, leading to the emergence of inflammophilic organisms, and increasing the virulence potential of the dysbiotic microbiota (Lamont and Hajishengallis, 2015).

Due to the essential role of the microbiota in host protection, the recovery of the microbial balance by the colonization of beneficial organisms associated with health appears as an attractive proposal in the prevention or as an adjuvant to the treatment of periodontitis (Raff and Hunt, 2012). Probiotics are “living microorganisms that, when given in adequate amounts, confer health benefits to the host (FAO/WHO, 2002).” These beneficial organisms may affect the microbiota by producing compounds with antimicrobial activity, competing for nutrients or adhesion sites, and altering the expression of virulence factors. They may also modulate the immune response, decrease permeability and bacterial translocation throughout epithelial barriers, and provide bioactive or regulatory metabolites (de Vrese et al., 2006; Mao et al., 2016; Matsubara et al., 2017; Albuquerque-Souza et al., 2018). Systematic reviews indicated that probiotic lactobacilli have the potential to improve periodontal conditions (Teughels et al., 2011; Matsubara et al., 2016). However, their mechanisms are still not fully elucidated.

*Aggregatibacter actinomycetemcomitans* has been associated with periodontitis formerly known as localized aggressive periodontitis (American Academy of Periodontology [AAP], 2001), now named periodontitis of the molar–incisor pattern (MIP) (Tonetti et al., 2018). The abundance of this species is 50 times higher in periodontal sites of MIP than in healthy subjects (Amado et al., 2020), and specially, the highly leukotoxic JP2 clone can trigger rapid periodontal destruction (Anders and Haubek, 2014). This species induces the production of inflammatory mediators by gingival epithelial cells (Umeda et al., 2012) and evades the immune response by internalizing in non-phagocytic cells and producing virulence factors such as a leukotoxin and a cytolethal distending toxin (Mayer et al., 1999; Kajiya et al., 2011; Shenker et al., 2016). *A. actinomycetemcomitans* is acquired in the oral microbiome early in life (Lamell et al., 2000), and the colonization of the oral cavity by this species, especially of the JP2 clone, may induce a precocious alveolar bone loss (Bueno et al., 1998; Haubek et al., 2004). Hence, the exposure of infected subjects to probiotics that limit *A. actinomycetemcomitans* overgrowth and modulate the inflammatory response may prevent the clinical alveolar bone loss and control its progression in *A. actinomycetemcomitans*-associated periodontitis. We have previously shown that *A. actinomycetemcomitans* biofilm formation and virulence may be impaired by probiotic lactobacilli (Ishikawa et al., 2021). However, little is known about the ability of probiotic strains to modulate the immune–inflammatory response induced by *A. actinomycetemcomitans*. Therefore, the purpose of this *in vitro* study was to evaluate the effect of two commercially available strains of lactobacilli on gingival epithelial cells (GECs) challenged by *A. actinomycetemcomitans*.

## Materials and Methods

### Study Design

The effects of lactobacilli probiotics on GECs infected with *A. actinomycetemcomitans* (strain JP2) were determined on cells viability, the adhesion of *A. actinomycetemcomitans* or probiotics, the release of cytokines in cells supernatants, and gene expression. All experiments consisted of six groups performed in triplicates: negative control (OBA-9 cells), positive control (OBA-9 cells co-cultured with *A. actinomycetemcomitans* JP2), probiotic controls (OBA-9 cells co-cultured with *Lactobacillus acidophilus* La5 or *Lacticaseibacillus rhamnosus* Lr32), and test groups (OBA-9 cells co-cultured with *A. actinomycetemcomitans* JP2 and *L. acidophilus* La5 or *L. rhamnosus* Lr 32). At least two independent assays were performed.

### Microorganisms and Culture Conditions

*Aggregatibacter actinomycetemcomitans* strain JP2 (Tsai et al., 1984), *L. rhamnosus* Lr32™ (DuPont™ and Danisco^®^, Madison, WI, United States), and *L. acidophilus* La5™ (CHR Hansen Holding A/S, Hørsholm, Denmark) were used for this study. Before the experiments, the strains were stored in 20% glycerol at −80°C.

*Aggregatibacter actinomycetemcomitans* was grown in 5% CO_2_ (microaerophilic conditions) at 37°C in tryptic soy agar with 0.6% yeast extract (TSYE, Difco Laboratories, Detroit, MI, United States) and in the brain–heart infusion broth (BHI; Difco). Lactobacilli were also cultivated under microaerophilic conditions in Lactobacilli MRS broth and agar (Lactobacilli MRS, Difco). Bacteria were grown in liquid media until the mid-log phase. Then, the suspensions were adjusted to an OD_490nm_ ∼ 0.3 for *A. actinomycetemcomitans*, corresponding to 1 × 10^8^ CFU/mL, and to an OD_590nm_ ∼ 0.9 for both lactobacilli, corresponding to 2 × 10^8^ CFU/mL.

### Cell Culture

Immortalized human gingival epithelial cells (OBA-9) (Kajiya et al., 2011) were cultured at 37°C in 10% CO_2_ in the serum-free keratinocyte medium (KSFM-Invitrogen, Carlsbad, CA, United States), supplemented with human recombinant epidermal growth factor (EGF), PenStrep GIBCO™ (penicillin: 10,000 units⋅mL^–1^/streptomycin: 10,000 μg⋅mL^–1^), and 25 μg/mL of amphotericin B (Invitrogen).

### Co-culture Assay

OBA-9 cells (∼2 × 10^5^ cells/well) were inoculated in 24-well tissue culture plates (Corning Inc., Corning, NY, United States) and incubated for 24 h to reach a semiconfluent monolayer (∼3 × 10^5^ cells/well) in KSFM. Before the infection, the wells were washed with phosphate-buffered saline (PBS) (pH 7.5, 0.8% NaCl). Mid-log *A. actinomycetemcomitans* culture in BHI broth was harvested by centrifugation, resuspended in antibiotic-free KSFM, and inoculated in OBA-9 cell monolayers at a multiplicity of infection (MOI) of 100:1 (bacteria/eukaryotic cell). Mid-log lactobacilli cultures in MRS broth were harvested by centrifugation, resuspended in antibiotic-free KSFM, and inoculated in OBA-9 cells at a multiplicity of infection (MOI) of 10:1 (bacteria/eukaryotic cell). After 2 h of incubation, cells were either collected or washed with PBS and fresh medium was added, completing 24 h of incubation.

### Cell Viability

Cell viability was estimated by trypan blue exclusion assay. Cells were released with Accutase^®^ Cell Detachment Solution (BioLegend), and cell counting was performed using the Countess Automated Cell Counter (Invitrogen).

### Adhesion Assay

After 2 h of incubation, non-adherent bacterial cells were removed by washing with PBS (pH 7.5, 0.8% NaCl). Then, the eukaryotic cells were subjected to osmotic lysis with 1 mL of sterile water for 20 min. Analysis of the number of adherent/internalized bacteria was determined by quantitative real-time PCR (qPCR), according to Ando-Suguimoto et al. (2014). Total DNA extraction was performed using the (Master Pure™ DNA Purification Kit, Epicenter, Madison, WI, United States).

Quantitative analysis was performed by comparing the CT from each sample with standard curve data obtained with template DNA of 16S rRNA *A. actinomycetemcomitans, L. acidophilus* La5, or *L. rhamnosus* Lr32 in recombinant plasmids [1500 bp of *16S rRNA* of each studied strain inserted in qPCR 2.1 TOPO TA^®^ vector, Invitrogen].

The reactions consisted of 5 μL of SYBR Green, 1 μL of template DNA, and 0.25 pmol of each species-specific stock solution primer (Table 1) to a final volume of 10 μL. The reaction was performed in 40 cycles at 95°C for 15 s, 65°C for 1 min, 81°C for 10 s, followed by two stages at 95°C for 15 s and 65°C for 1 min, and one final stage at 0.5–95°C for 10 s. The number of microorganisms was calculated assuming six copies of 16S rRNA/chromosome for *A. actinomycetemcomitans*, five copies of *L. rhamnosus* Lr32, and four copies of *L. acidophilus* La5^1^. Data were expressed as the number of cells/well.

**TABLE 1:** 16S rRNA primer sequences for a quantitative analysis by qPCR of *Aggregatibacter actinomycetemcomitans* and lactobacilli.

Bacterial group	Oligonucleotide 5′ - 3′	Reference
*A. actinomycetemcomitans*	F: 5′-ATTGGGGTTTAGCCCTGGT-3′ R: 5′-GGCACAAACCCATCTCTGA-3′	Rudney et al., 2003
Lactobacillus sp.	F: 5′-AGCAGTAGGGAATCTTCCA-3 R: 5′-CACCGCTACACATGGAG-3′	Aroniadis and Brandt, 2014

### Quantification of Inflammatory Mediators

Levels of interleukin (IL) 1β, IL-10, C-X-C motif chemokine ligand 8 (CXCL-8), and *g*ranulocyte–macrophage colony-stimulating factor (GM-CSF) were determined in cells supernatants of 2- and 24-h co-culture assays by ELISA (BD Systems^®^) according to the manufacturer’s recommendations. The reading was performed on a microplate reader (Model 680, cat. 1681000, BioRad, Tokyo, Japan) adjusted at a wavelength of 450 nm, and data were expressed as pg/mL.

### Gene Expression

The relative gene expression of OBA-9 cells was determined by reverse transcription followed by quantitative real-time PCR (RT-qPCR). After 24 h of co-culture, OBA-9 cells were lysed, and total RNA was extracted using RNeasy kit (Qiagen, Valencia, CA, United States) and reverse-transcribed into cDNA using SuperScript VILO MasterMix (Invitrogen, Waltham, MA, United States). Relative expression levels were evaluated by PCR in a TaqMan Gene Expression Master system (Applied Biosciences, Foster City, CA, United States), using 200 ng of cDNA and TaqMan primers and probes for *TLR2* (Hs00152932_m1), *TLR4* (Hs01060206_m1), *NLRP3* (Hs00918082_m1), *BCL2* (Hs00608023_m1), *BAX* (Hs00180269_m1), and *GAPDH* (Hs02758991_gl). The qPCR comprised an initial step of 50°C for 2 min, 95°C for 10 min, followed by 40 cycles at 95°C for 15 s and 50°C for 1 min using the Step One Plus System (Applied Biosciences). All data were normalized to GAPDH transcript levels in the same cDNA set, and relative expression analysis was performed by the 2^–ΔΔCT^ method (Pfaffl, 2001).

### Apoptosis

The experiment was carried out with Annexin V as described by the manufacturer (FITC Annexin V apoptosis detection kit, BD Pharmingen, United States). After incubation of OBA-9 cells with *A. actinomycetemcomitans* and/or lactobacilli for 24 h, the plates were centrifuged, the supernatant was removed, and 500 μL of binding buffer with FITC and Ghost Dye Red (Tonbo Biosciences, San Diego, CA, United States) was added and then incubated for 20 min at room temperature away from light. Then, the plates were centrifuged, the supernatant was removed, 500 μL of binding buffer was added to the wells, and the cells were detached using a cell scraper (Kasvi, São José dos Pinhais, PR, Brazil). The cells were analyzed by a cytometry system (BD FACSCanto Flow Cytometry Systems, Piscataway, NJ. United States). As a positive control, camptothecin (6 μM) was added to the wells 6 h before the analysis.

### Statistical Analysis

Data were reported as mean ± standard deviation (SD) from at least two independent experiments. A one-way ANOVA test followed by Tukey’s multiple comparison test was applied for statistical analyses. Statistical significance was set at *p* < 0.05 (BioStat Software 5.3, Belém, Brazil).

## Results

### Pathogen and Lactobacilli Alter Cell Viability After 24 h of Incubation

**FIGURE 1:**
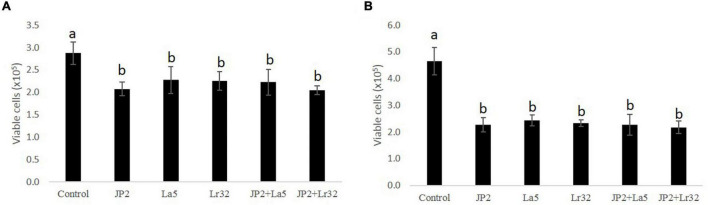
Cell viability determined by trypan blue exclusion in OBA-9 cells mono-infected with *A. actinomycetemcomitans* (JP2) and/or *L. acidophilus* La5 (La5) or *L. rhamnosus* Lr32 (L32) for 2 h **(A)** and 24 h **(B)**. Statistical test: ANOVA/Tukey, different letters mean *p* < 0.05. In the y-axis is the number of viable OBA-9 cells (x10^5^). Control corresponds to non-infected GECs.

The cell viability data of OBA-9 cells after interaction with *A. actinomycetemcomitans* and/or probiotics after 2 and 24 h are shown in Figures 1A,B reduction in cell viability was observed for all experimental groups at 2 and 24 h compared with the uninfected control cells (*p* < 0.05).

### Lactobacilli Reduced Adhesion of *Aggregatibacter actinomycetemcomitans* to Gingival Epithelial Cells

**FIGURE 2:**
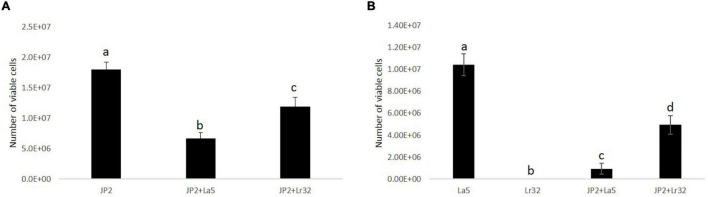
*A. actinomycetemcomitans* (JP2) **(A)** and lactobacilli **(B)** adherent/internalized into OBA-9 mono-infected with *A. actinomycetemcomitans* (JP2), *L. acidophilus* La5 (La5), or *L. rhamnosus* Lr32 (L32) and co-infected with the pathogen and *L. acidophilus* La5 (JP2 + La5) or *L. rhamnosus* Lr32 (JP2 + L32) for 2 h. Data are expressed as the number of bacterial cells/well, as estimated by qPCR. ANOVA/Tukey, and different letters indicate statistical differences among groups (*p* < 0.05).

The quantitative analysis data indicated that *A. actinomycetemcomitans* and *L. acidophilus* La5 adhered/invaded OBA-9 cells, while mono-infection of OBA-9 cells with *L. rhamnosus* Lr32 yielded no detectable adherent lactobacilli. Both lactobacilli significantly reduced *A. actinomycetemcomitans* adhesion/invasion to OBA-9 cells (*p* < 0.05). Co-infection of OBA-9 cells with *A. actinomycetemcomitans* and *L. acidophilus* La5 yielded lower levels of the probiotic than the mono-infection. However, the co-infection of GECs with *A. actinomycetemcomitans* and *L. rhamnosus* Lr32 favored the adhesion of the lactobacilli (Figure 2).

### Lactobacilli Altered Inflammatory Mediators’ Profile of Gingival Epithelial Cells Challenged With *Aggregatibacter actinomycetemcomitans*

**FIGURE 3:**
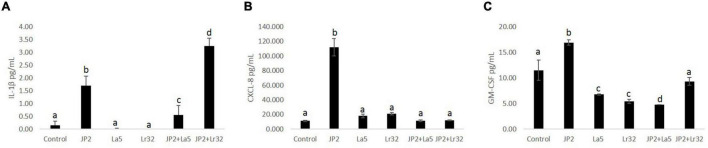
Levels of IL-1β **(A)**, CXCL-8 **(B)**, and GM-CSF **(C)** (pg/mL) determined by ELISA in cells supernatants of OBA-9 mono-infected with *A. actinomycetemcomitans* (JP2) and/or *L. acidophilus* La5 (La5) or *L. rhamnosus* Lr32 (L32) after 24 h of incubation. Control corresponds to non-infected GECs. ANOVA/Tukey; different letters indicate statistical differences among groups (*p* < 0.05).

Levels of IL-1β, IL-10, CXCL-8, and GM-CSF were determined in the co-culture supernatant. No detectable levels of any studied mediator were observed in the supernatant after 2 h of co-culture with either tested bacteria, and IL-10 was not detected even after 24-h incubation (data not shown). None of the lactobacilli induced significant levels of any studied mediator. *A. actinomycetemcomitans* promoted a slight release of IL-1β after 24 h of interaction, which was attenuated by the co-infection with *L. acidophilus* La5, but increased with *L. rhamnosus* Lr32 (*p* < 0.05). CXCL-8 production was highly induced by the pathogen, and both probiotics attenuated its production (*p* < 0.05). *Aggregatibacter actinomycetemcomitans* also induced the release of GM-CSF by OBA-9 cells, which was reduced by the lactobacilli, especially by *L. acidophilus* La5 (*p* < 0.05) (Figure 3).

### Lactobacilli Modulate the Transcription of *TLR2, NLRP3*, and *BCL-2*

**FIGURE 4:**
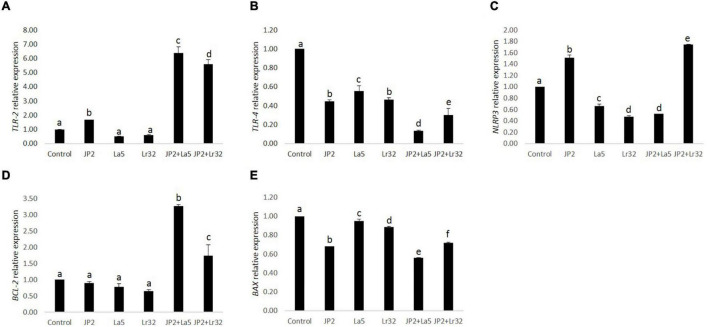
Relative transcription of *TLR-2* **(A)**, *TLR-4* **(B)**, *NLRP3* **(C)**, *BCL-2* **(D)**, and *BAX* **(E)** in GECs mono-infected with *A. actinomycetemcomitans* (JP2) and/or *L. acidophilus* La5 (La5) or *L. rhamnosus* Lr32 (Lr32) for 24 h. Data on target genes were normalized to mRNA levels of the GAPDH reference gene (internal control). Control corresponds to non-infected GECs. ANOVA/Tukey, and different letters indicate statistical differences among groups (*p* < 0.05).

The challenge of GECs with *A. actinomycetemcomitans* was found to upregulate *TLR-2* mRNA levels, which were magnified when *A. actinomycetemcomitans*-infected cells were co-infected with any of the studied lactobacilli (*p* < 0.05). Nevertheless, *TLR-4* was downregulated by *A. actinomycetemcomitans*, and the probiotics further reduced *TLR-4* mRNA levels. Transcription of the *NLRP3* was positively regulated by *A. actinomycetemcomitans* and negatively regulated by *L. acidophilus* La5. However, *L. rhamnosus* Lr32 further increased the expression of *NLRP3.* In addition, the mRNA levels of the anti-apoptotic gene *BCL-2* were positively regulated when pathogen-infected epithelial cells were co-cultured with the probiotics when compared to uninfected cells (control) or to cells challenged by *A. actinomycetemcomitans* (*p* < 0.05). Moreover, the transcription of *BAX*, encoding a pro-apoptotic factor, was negatively regulated when epithelial cells were challenged with *A. actinomycetemcomitans* and probiotics when compared to uninfected cells (control) (*p* < 0.05) (Figure 4).

### Apoptosis

Infection with *A. actinomycetemcomitans* significantly increased the number of apoptotic cells (Annexin V positive, Ghost Dye Red negative) after 24 h of interaction compared with the negative control (*p* < 0.05). Furthermore, none of the lactobacilli alone interfered with cell viability compared to the negative control. Nevertheless, in the co-culture of *A. actinomycetemcomitans* with any of the lactobacilli, a significant decrease in apoptotic and necrotic cells (*p* < 0.05) was observed.

## Discussion

*Aggregatibacter actinomycetemcomitans* initiates an aberrant inflammatory response in the gingival periodontal tissues through the interaction of its virulence factors with host cells (Fives-Taylor et al., 1999). The first cells that subgingival organisms face are the gingival epithelium, which acts as a physical barrier and plays an important role in innate immune response (Groeger and Meyle, 2019).

Using a co-culture model, our data showed that *A. actinomycetemcomitans* adhesion to GECs was reduced by both tested lactobacilli, although the inhibition of the adhesion promoted by strain *L. acidophilus* La5 was more pronounced than that by *L. rhamnosus* Lr32. Furthermore, *L. acidophilus* La5 was able to adhere to GECs, and this adhesion was reduced by the pathogen, suggesting that this probiotic could adhere to the oral epithelium surface and compete with the pathogen for adhesion sites. Alternatively, other competitive exclusion mechanisms, such as the production of acids, bacteriocins, or oxidative compounds, also may be involved (Cáceres et al., 2011; Teughels et al., 2011). On the other hand, *L. rhamnosus* Lr32 only yielded detected levels of adherent bacteria in GECs co-infected with *A. actinomycetemcomitans*, suggesting that the pathogen bound to GECs could act as a bridge, aggregating with *L. rhamnosus*.

The antimicrobial activity of lactobacilli has been reported. Previous data reported that lactobacilli suppressed *in vitro* growth of periodontal pathogens in a strain- and a species-specific way (Koll-Klais et al., 2005). Similarly, *in vitro* data demonstrated that lactobacilli reduced the interaction of *P. gingivalis* to epithelial cells (Albuquerque-Souza et al., 2018) and reduced *P. gingivalis* biofilm formation but did not affect commensal streptococci (Ishikawa et al., 2020).

Our group has also shown that secreted products of *L. acidophilus* La5 were able to reduce biofilm formation by *A. actinomycetemcomitans* and pre-formed biofilm, whereas transcription of cytolethal distending toxin (*cdtB*) and leukotoxin (*ltxA*) was downregulated by cell-free pH-neutralized supernatants of *L. acidophilus* La5 and *L. rhamnosus* Lr32 (Ishikawa et al., 2021). The levels of *ltxA* and *cdtB* were also analyzed in another study, and both genes were downregulated in a time-dependent way when *A. actinomycetemcomitans* was cultured with *Lactobacillus salivarius* and *Lactobacillus gasseri* cell-free supernatants (Nissen et al., 2014).

Cell response to microbial challenges is mediated by the recognition of bacterial pathogen-associated molecular patterns (PAMPS) by pattern recognition receptors (PPRs), located in the cell membrane and in the cytosol. Cell membrane receptors, such as toll-like receptors (TLRs), represent one of the first mechanisms of immune defense against invading organisms (Groeger and Meyle, 2019). A major consequence of the activation of surface TLRs, such as TLR-2 and TLR-4, is the induction of pro-inflammatory cytokines and chemokines, which play an essential role in innate and adaptive immune responses (Beklen et al., 2008; Oliveira-Nascimento et al., 2012; Groeger and Meyle, 2019).

In this study, the expression of *TLR-2* and *TLR-4* was evaluated in cells challenged with *A. actinomycetemcomitans* and with the probiotics. Our data indicated that *A. actinomycetemcomitans* challenged and/or the probiotics downregulated *TLR-4* expression, and this reduction was more pronounced when the cells were co-infected with *L. acidophilus* La5. On the other hand, *A. actinomycetemcomitans* challenge promoted a slight upregulation of *TLR-2*, whereas mono-infection with probiotics did not alter baseline *TLR-2* mRNA levels. However, the combination of the challenge with the pathogen and the probiotics led to a statistically significant increase in TLR-2 expression.

TLR-2 is highly expressed in the basal layer of the gingival epithelium, whereas its levels are lower in the superficial layers that are more exposed to microorganisms (Groeger and Meyle, 2019). It is well known that lipoproteins and teichoic acid on the cell surface of lactobacilli are recognized by TLR-2 (Sengupta et al., 2013). In addition, certain lactobacilli such as *L. plantarum* BFE 1685 and *L. rhamnosus* GG can lead to TLR-2, but not to TLR4, upregulation in intestinal epithelial cells, but this effect is reduced when the cells were challenged with an enteropathogen (Vizoso et al., 2009). It has been shown that the probiotic anti-inflammatory effects are dependent on TLR-2 recognition (Sun et al., 2017). Thus, the enhancement in *TLR2* expression promoted by lactobacilli in GECs would lead to a decrease in the production of inflammatory mediators in cells challenged by the pathogen.

Indeed, the co-infection of *A. actinomycetemcomitans-*challenged cells with the probiotics resulted in reduced levels of certain studied factors. The interaction of *A. actinomycetemcomitans* with GECs resulted in the activation of epithelial cells, which produced CXCL-8 and GM-CSF, as previously shown (Uchida et al., 2001; Stathopoulou et al., 2010; Umeda et al., 2012), whereas exposure to the probiotics attenuated the response of GECs, which produced lower levels of CXCL-8 and GM-CSF. This altered response cannot be regarded only as the reduced interaction of GECs with the pathogen, since both probiotics reduced adherence of *A. actinomycetemcomitans* to GECs but acted differently in altering the cell response. *L. acidophilus* La5 modulated the inflammatory response against the challenge with *A. actinomycetemcomitans* by decreasing the release of CXCL-8, GM-CSF, and IL-1β, whereas *L. rhamnosus* Lr32 reduced the levels of CXCL-8 and GM-CSF but increased the production of IL-1β.

We have shown that *A. actinomycetemcomitans per se* can induce IL-1β production, as seen in cell supernatant of GECs (Figure 3). The secretion of active IL-1β requires cytosolic sensing by intracellular receptors, including NLRP3 (Figure 4), which leads to the formation of multiprotein cytoplasmic complexes, called inflammasomes. These complexes activate caspase-1, which results in the release of mature IL-1β (Franchi et al., 2012). Previous data indicated that *A. actinomycetemcomitans* can activate the inflammasome in leukocytes and macrophages, particularly due to the production of the cytolethal distending toxin (Belibasakis and Johansson, 2012; Ando-Suguimoto et al., 2014; Shenker et al., 2015). On the other hand, the response to *A. actinomycetemcomitans* challenge for 8 h on primary gingival epithelial cells did not indicate inflammasome activation (Ando-Suguimoto et al., 2020), although there are no data on longer incubation periods.

Probiotics may attenuate the secretion of IL-1β, as our data indicated when *L. acidophilus* La5 was tested when combined with *A. actinomycetemcomitans* challenge in GECs. However, the addition of *L. rhamnosus* Lr32 to GECs challenged with the pathogen resulted in increased production of IL-1β. The increased IL-1β levels in cells co-infected with *A. actinomycetemcomitans* and *L. rhamnosus* Lr32 suggest that this strain may act synergistically with the pathogen to activate the inflammasome. This hypothesis is corroborated by our observation of the upregulation of *NRLP3*, encoding the intracellular receptor for NRLP3 inflammasome activation, when the GECs were co-cultured with *A. actinomycetemcomitans* and *L. rhamnosus* Lr32. It seems that *L. rhamnosus* Lr32 may not activate the inflammasome itself but may increase inflammasome activation under certain conditions. The increased production of IL-1β promoted by *L. rhamnosus* Lr32 in cells challenged with *A. actinomycetemcomitans* is similar to what was observed in the co-infection of the same probiotic with *P. gingivalis* on GECs (Albuquerque-Souza et al., 2018). Other strains of *L. rhamnosus* were also shown to activate inflammasome, which would favor pathogen elimination (Korpela et al., 2012), but may induce further damage in diseases associated with an exacerbated inflammatory response such as periodontitis (Ebersole et al., 2013).

CXCL-8 is a chemoattractant produced by several types of cells to attract neutrophils and other defense cells to the infection site (Russo et al., 2014). Despite its importance in defense, the production of CXCL-8 in already inflamed tissues, such as in periodontitis, may contribute to the loss of the connective tissue and the destruction of the alveolar bone (Finoti et al., 2017). CXCL-8 production by gingival epithelial cells is dependent on the composition of the microbial community (Belibasakis et al., 2013), and data on CXCL-8 levels in the gingival crevicular fluid of periodontitis patients are still contradictory (Finoti et al., 2017). Some probiotic lactobacilli may lead to increased (Vizoso et al., 2009) or decreased (Stamatova et al., 2010) CXCL-8 production *in vitro*, although clinically controlled trials of periodontal patients using probiotics as an adjunctive therapy to conventional treatment indicated decreased levels of CXCL-8 in the gingival crevicular fluid (Twetman et al., 2009).

Our data showed that both studied lactobacilli were able to decrease GM-CSF released by GECs co-cultured with *A. actinomycetemcomitans*. GM-CSF is an important cytokine in the survival, proliferation, and differentiation of macrophages and neutrophils (Hamilton, 2008), and it is known to be upregulated after the interaction between *A. actinomycetemcomitans* and gingival epithelial cells (Umeda et al., 2012), which results in the recruitment and differentiation of immune cells, such as macrophages, which are responsible for a pro-inflammatory response involving antigen presentation, phagocytosis, and release of IL-1β (Gholizadeh et al., 2017). A previous study has demonstrated that a mixture of three probiotics (*Enterococcus faecalis*, *Bifidobacterium longum*, *and L. acidophilus*) decreased the levels of GM-CSF produced by gastric mucosal epithelial cells infected with *Helicobacter pylori* (Yu et al., 2015). *L. rhamnosus L34* and *L. casei L39* conditioned media decreased the production of GM-CSF by *Clostridium difficile*-stimulated HT-29 intestinal epithelial cells, which is the main cause of hospital-acquired diarrhea and colitis (Boonma et al., 2014). Thus, the decrease in GM-CSF levels seems beneficial in inflamed mucosa surfaces, including in periodontitis. Increased GM-CSF levels were associated with an allele of increased susceptibility to aggressive periodontitis (Harris et al., 2020), whereas the destruction of the alveolar bone was dependent on GM-CSF in a mouse experimental model (Lam et al., 2015).

**FIGURE 5:**
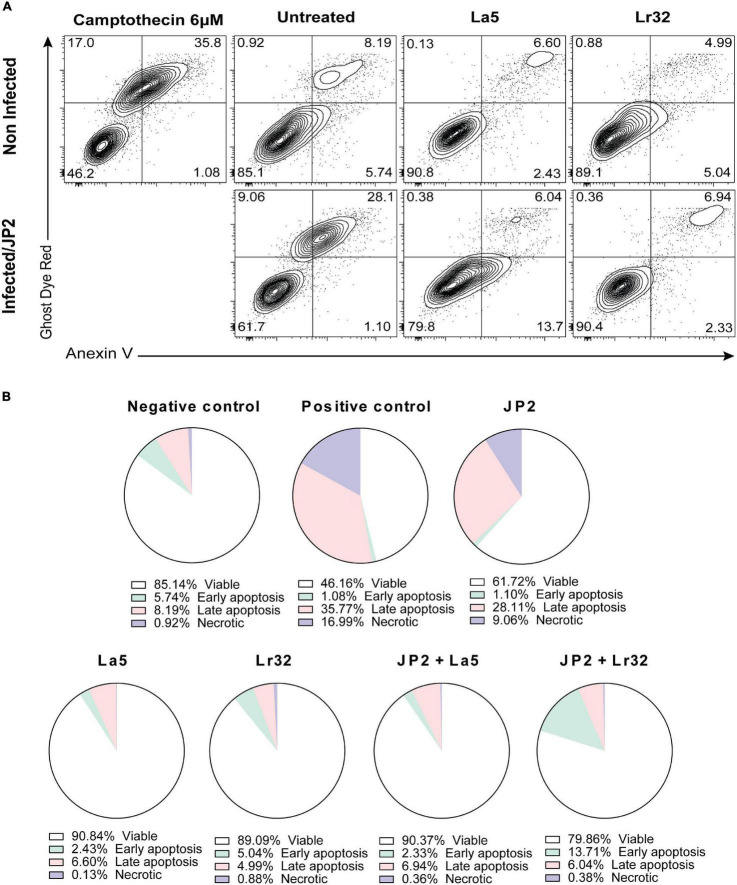
Cytotoxicity by *A. actinomycetemcomitans* is mediated through apoptosis in gingival epithelial cells (GECs). **(A)** GECs were treated with *A. actinomycetemcomitans* at an MOI of 1:100 for 24 h and/or *L. acidophilus* La5 or L. *rhamnosus* Lr32 at an MOI 1:10. Camptothecin 6 uM was used as the positive control. The cells were stained with Annexin V and Ghost Dye Red and then analyzed using flow cytometry. Numbers indicate the percentage of cells in each panel. **(B)** Pie chart with the percentage of viable, early apoptosis, late apoptosis, and necrotic cells.

Since we observed a reduction in cell viability of GECs co-infected with *A. actinomycetemcomitans* and/or lactobacilli, we analyzed the expression of genes involved in apoptosis, of the anti-apoptotic gene *BCL-2*, and of the pro-apoptotic *BAX.* Co-culture with *A. actinomycetemcomitans* did not alter the transcription of *BCL2* and reduced mRNA levels of *BAX*, whereas the probiotics altered their expression. Both lactobacilli induced the transcription of *BCL2* in *A. actinomycetemcomitans-*challenged GECs, and *L. acidophilus* LA5 also decreased the transcription of *BAX*, suggesting that the probiotics would protect epithelial cells from apoptosis, confirmed by flow cytometry analysis (Figure 5). Other studies reported the effectiveness of probiotic lactobacilli in altering the expression of genes involved in apoptosis of hepatocytes *in vitro* (Sharma et al., 2011) and *in vivo* experimental models (Chen et al., 2018).

In summary, this evidence suggests that lactobacilli, especially *L. acidophilus* La5, can modulate the inflammatory response mediated by *A. actinomycetemcomitans* in GECs. The evaluated probiotics strains were able to reduce pathogenic bacteria adhesion to host cells, alter the release of inflammatory chemo/cytokines in a strain-specific way, and alter the pathogen’s recognition profile and influence the production of the anti-apoptotic gene. These results illustrate that probiotic modulatory mechanisms should be evaluated toward controlling the inflammatory response followed by chronic inflammation. A more detailed understanding of their properties could result in their use as an adjunctive therapy for periodontal disease.

## Data Availability Statement

The original contributions presented in the study are included in the article/supplementary material, further inquiries can be directed to the corresponding author.

## Author Contributions

MM conceptualized and designed the project, planned the assays, and wrote the manuscript. MB planned and ran the assays and helped to wrote the manuscript. DK, EA-S, NS, GA-S, and KI helped with the sample analyses. All authors revised the manuscript.

## Conflict of Interest

The authors declare that the research was conducted in the absence of any commercial or financial relationships that could be construed as a potential conflict of interest.

## Publisher’s Note

All claims expressed in this article are solely those of the authors and do not necessarily represent those of their affiliated organizations, or those of the publisher, the editors and the reviewers. Any product that may be evaluated in this article, or claim that may be made by its manufacturer, is not guaranteed or endorsed by the publisher.

## Footnotes

1
https://rrndb.umms.med.umich.edu/genomes
